# Poor obstetric outcomes in women with takayasu arteritis: a retrospective cohort study

**DOI:** 10.1007/s00296-024-05538-z

**Published:** 2024-02-16

**Authors:** Erdal Bodakçi, Döndü Üsküdar Cansu, Cengiz Korkmaz

**Affiliations:** 1 Division of Rheumatology, Department of Internal Medicine, Eskisehir State Hospıtal, Eskisehir, 26100 Turkey; 2 Division of Rheumatology, Faculty of Medicine, Department of Internal Medicine, Eskişehir Osmangazi University, 26480 Eskişehir, Turkey

**Keywords:** Takayasu arteritis, Pregnancy, Obstetric, Outcomes

## Abstract

The objective of this study was to assess the pregnancy outcomes in a cohort of patients who experienced pregnancies before and/or after being diagnosed with Takayasu’s arteritis (TA). The present investigation encompassed a total of 88 pregnancies seen in a cohort of 35 patients who met the criteria outlined by the American College of Rheumatology in 1990 for the classification of Takayasu arteritis (TA). Pregnancies were classified into two categories. 1. Pregnancies that occurred before the diagnosis (pre-d or pre-TA) 2. Pregnancies that happened following a diagnosis (post-d or post-TA). Fifty-nine pregnancies (67.0%) occurred in 21 TA patients before the diagnosis with and a complication rate of 15.2%, and twenty-nine pregnancies (33.0%) occurred in 14 patients concomitant with or after TA diagnosis and complication rate 100%. Although the hypertension rate was higher in the pre-d group than in the post-d group, it was not significant (32.2% vs. 10.3%, *p* = 0.160). However, preeclampsia (20.6% vs. 0%, *p* = 0.001), low birth weight (27.5% vs. 1.6%, *p *= 0.001), and prematurity (24.1% vs. 1.6%, *p* = 0.035) were observed more frequently in the post-d group compared to the pre-d group. The frequency of abortions and in-utero deaths were similar in both groups (*p* > 0.05). Patients with hypertension had significantly higher rates of preeclampsia (*p* = 0.003), preterm birth (*p* = 0.036), low birth weight (*p* = 0.250), abortion (*p* = 0.018), in utero death (*p* = 0.128), and cesarean section (*p* = 0.005) than those without hypertension. Renal artery involvement was detected in 15 (42.8%) patients. All patients with renal artery involvement had hypertension, and they had significantly more pregnancy complications than the other group (*p* = 0.001). TA negatively affects pregnancy outcomes. A good control of arterial hypertension before conception and during pregnancy is critical to improve both maternal and fetal outcomes. In addition, detecting renal artery stenosis before pregnancy is important in reducing possible negative pregnancy outcomes.

## Introduction

Takayasu arteritis (TA) is a chronic granulomatous large vessel vasculitis that primarily affects the aorta and its primary branches [1]. TA usually starts in women of childbearing age, and reproductive health is therefore an important issue[2]. Pregnancy is a more common occurrence among patients with TA than with other systemic vasculitides [3]. Pregnancy can affect the diagnosis, management and outcome of TA. During pregnancy, the maternal body experiences alterations to its circulatory system to guarantee appropriate oxygenation and nutrition for the developing fetus, additionally, it serves to safeguard the mother from the adverse impacts of reduced venous return caused by uterine compression and blood loss during delivery [4]. TA is characterized by the thickening of the arterial wall, eventually leading to stenosis, obliteration, or aneurysm formation. The inflammation and ischemia of the organs supplied by the affected arteries can lead to clinical manifestations. This can have a negative impact on both maternal health and fetal growth [5]. To date, there is limited research regarding the pregnancy outcome of TA patients with inconsistent findings present in the literature [6–9]. New findings indicate that expectant mothers with TA are more likely to experience hypertension, miscarriage, and IUGR in their infants, with this additional risk linked to elevated disease activity during pregnancy [5]. However, it has been demonstrated by other studies that the activity of TA disease did not have an association with negative pregnancy outcomes [6, 10].

The aim of this study was to evaluate maternal complications and fetal outcomes in patients with TA pregnancies, to assess patients before and after diagnosis and to compare pregnancies.

## Material and methods

In a retrospective study, we reviewed data from 88 pregnancies in 35 patients treated with TA and followed at the Faculty of Medicine, Division of Rheumatology, Eskişehir Osmangazi University, between January 2000 and October 2019. Patients who were diagnosed with Takayasu arteritis, according to the classification criteria established by the American College of Rheumatology, have been included in this study. [11]. The exclusion criteria encompassed nulliparity, those under the age of 18, the presence of additional concomitant vasculitis, and systemic disorders such as diabetes mellitus, systemic lupus erythematosus, antiphospholipid syndrome, rheumatoid arthritis, chronic renal disease, and heart failure. Additionally, participants who experienced loss of follow-up were also excluded.

The pregnancies of the patients were divided into two categories based on the date of their TA diagnosis. The first group is pregnancies that occurred before the diagnosis (pre-d or pre-TA), this group includes patients who had pregnancies before TA was diagnosed and those who are not pregnant after diagnosis, the second group is those diagnosed with TA after diagnosis or during pregnancy (post-d or post-TA), in this group are those who did not have a pregnancy before TA diagnosis. The diagnosis was based on clinical findings during pregnancy. After delivery, magnetic resonance imaging or computed tomography angiography was performed to provide a complete evaluation and classified into five categories according to Hata et al.'s angiographic classification [12].

The demographic, clinical, and laboratory data and pregnancy histories of the patients were evaluated. Patients who gave birth in our hospital and whose data were accessed were included. The study obtained maternal medical records, pregnancy follow-up, mode of delivery, complications, and neonatal outcomes for each patient from hospital medical records. Furthermore, at research commencement, for the reliability and accuracy of the records, a questionnaire was also administered to the patients and confirmed. A standardized questionnaire was administered to all participants in order to evaluate the basic characteristics of the individuals, including age, gestational age, and parity. The questionnaire was administered either at the outpatient clinical appointment or by telephone. The patient's self-reported information was used to assess maternal outcomes and obstetric problems in this study, as determined by the questions asked. We inquired about prior diagnoses of arterial hypertension, preeclampsia, eclampsia, infection, renal and cardiac insufficiency, as well as other symptoms indicative of TA, such as fever, syncope, claudication, carotidynia, and arthritis. Additionally, the questionnaire included inquiries regarding the method of delivery (vaginal or cesarean), occurrences of spontaneous abortion (defined as those happening before the 12th week of gestation), as well as detailed data on fetal outcomes such as IUGR and fetal weight below the 10th centile for the corresponding gestational age [13], prematurity (delivery before 37 weeks of gestation) and perinatal mortality (deaths occurring between the 28th week of pregnancy and the seventh day after birth). Intrauterine death (IUD): a baby born without any evidence of life beyond 28 weeks of gestation [14]. The National Institutes of Health (NIH) standards were used to detect disease activity and calculate damage [15]. The assessment of disease activity utilizes the NIH definitions, which comprise four components: systemic features (with no other identified cause); elevated erythrocyte sedimentation rate (ESR) or C-reactive protein (CRP) level; features of vascular ischemia or inflammation (such as extremity claudication, diminished or absent pulse, bruits, pain over large vessels, or asymmetric blood pressure); and new vascular lesion (s) on imaging studies, i.e., new stenosis or new dilatation (15). An NIH score of ≤ 1 (inactive) or ≥ 2 was considered an active disease. The ethics committee at Eskişehir Osmangazi University approved our the study (Approval number: 17.12.2019/06), and informed consent was obtained from all participants.

### Statistical analysis

Categorical variables were expressed as percentages (%) and frequencies, and continuous variables with normal distributions were expressed as mean ± standard deviation (SD). Variables that did not conform to a normal distribution were reported as median and interquartile range (IQR). If they contained categorical data, they are expressed as percentage (%) and frequency (*n*). Differences between continuous variables and categorical data were tested using Student’s *t*-test (if data was distributed normally) or the Mann–Whitney test and Chi-square test. A two-sided P value of < *0.05* was considered significant. Analysis was performed with the SPSS software (version 26).

## Results

### Demographic data and clinical characteristics

**Fig. 1:**
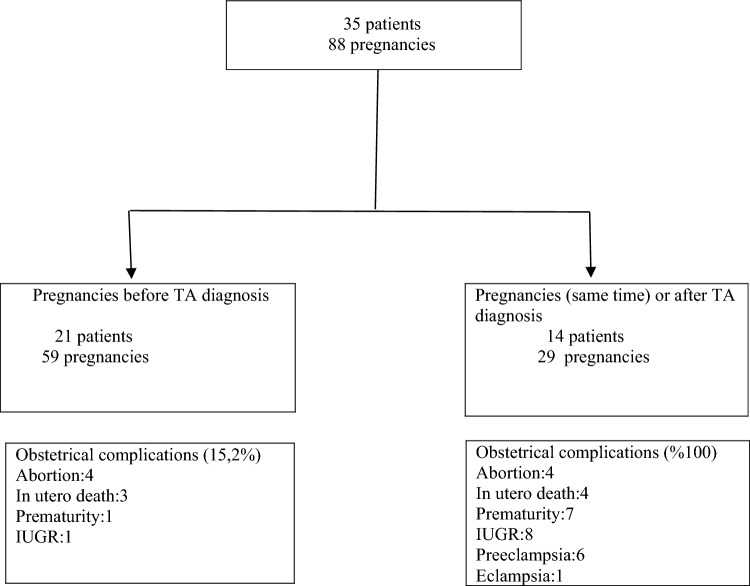
Flow chart showing the pregnancies and patients included in the study. TA Takayasu arteritis

The main clinical and demographic characteristics of the 88 pregnancies in 35 women with TA are presented in Table 1 and Fig. 1. Twenty-one of these patients were diagnosed pre-d and had 59 pregnancies, while 14 were post-d and had 29 pregnancies. The mean age was 41.0 ± 7.35 years and the median disease duration was 10 (IQR 1–20) years. The median age at TA diagnosis was 30 (20–48). Age at pregnancy onset, median (IQR) years 30 (19–38). The number of patients in primigravida was 3(8.5%), and multigravida was 32 (91.5%). The delivery mode included vaginal delivery in 24(68.6%) and cesarean section in 11(31.4%) patients. Patients most frequently had type V angiographic findings (40%) according to the Numano classification [12].

**Table 1:** Characteristics of the 35 patients with Takayasu’s arteritis

Demographics	*n* = 35
Age, year, mean ± SD	41.0 ± 7.35
Age at Takayasu’s arteritis diagnosis, median [range]	30 [20–48]
Age at pregnancy onset, median (IQR) years [range]	30 [19–38]
Disease duration, y, median [range]	10 [1–20]
Primigravida, *n* (%)	3 (8.5)
Multigravida,*n* (%)	32 (91.5)
*Delivery*	
Vaginal, *n* (%)	24 (68.6)
Caesarean, *n* (%)	11 (31.4)
*Numano classification, n(%)*	
Type I	10 (28.6)
Type II	7 (20)
Type III	2 (5.7)
Type IV	2 (5.7)
Type V	14 (40)
*Cardiovascular risk factors*	
Smoking *n* (%)	4 (14.2)
Hypertension, *n* (%)	22 (62.9)
Dyslipidemia *n* (%)	4 (11.4)
*Previous treatment,n*	
Steroids, *n* (%)	23(85.1)
Azathioprine, *n* (%)	14 (51.8)
Methotrexate, *n* (%)	4 (14.8)
Cyclophosphamide, *n* (%) 4 (14.8)	
TNF-α antagonist, *n* (%)	5 (18.1)
Aspirin, *n* (%)	16 (59.2)
Clopidogrel, *n* (%)	3 (11.1)
Vascular stent or surgery, *n* (%)	5 (18,1)
*Medicines during pregnancy, n*	
Steroids, *n* (%)	25 (83.3)
Azathioprine, *n* (%)	10 (33.3)
Aspirin (75–100 mg/days)	20 (66.6)
Clopidogrel (75 mg/days)	2 (6.6)
Antihypertensive agents	18 (72.0)
Vascular stent or surgery	0 (0)

### Vascular involvement

The abdominal and renal arteries were involved in 51.4% and 14.2% of the patients with TA, respectively. Major cardiovascular risk factors were a history of hypertension (62.9%). Deep vein thrombosis, pulmonary embolism, cerebrovascular events, and heart failure did not develop in any pregnancy.

### Treatment

Twenty-three patients were treated with steroids (85.1%) before pregnancy, 14 with azathioprine (51.8%), 4 with methotrexate (14.8%), 5 with TNF-α antagonist (3 with infliximab, 2 with adalimumab, 18.5%) and 4 with cyclophosphamide (14.8%). Twenty-five patients were treated with steroids (83.3%) during pregnancy,and 10 with azathioprine (33.3%). Steroids were restarted in 10 patients, azathiopurine in 5, and infliximab in 2, depending on disease activity after pregnancy.

### Pregnancy outcomes

The most common maternal complication was hypertension (62.9%). This type of hypertension was more frequent in women with pre-pregnancy renal artery involvement than in those without 58.3% (7/12) versus 41.6% (5/12), (*p* = 0.032). During pregnancy, 10 (28.5%) patients had worsening hypertension. All of these patients had renal and abdominal artery involvement. Pre-d and post-d groups are compared in Table 2. Accordingly, preeclampsia (20.6%), eclampsia (3.4%), prematurity (24.1%), and IUGR (27.5%) were more common in the post-d group than in the pre-d group. Abortion and in-utero death were similar in both groups. In terms of number of pregnancies, there were 2 patients with one pregnancy, 17 with two pregnancies, 11 with three pregnancies, 2 with four pregnancies and 2 with five pregnancies. Three patients were diagnosed with TA during pregnancy, and 5 patients were diagnosed with TA after pregnancy. One patient was classified at the time of the diagnostic evaluation as having had a premature birth, while another had a low birth weight. One patient who was diagnosed after pregnancy had a child with cerebral palsy due to insufficient oxygenation during birth.

**Table 2:** Comparison between the pregnancy outcomes in the pre-TA and post-TA groups

Variables	pre-TA Group 59 pregnancies	post-TA Group 29 pregnancies, *n*, %	*p* valves
Preeclampsia	0 (0)	6 (20.6)	0.001
Eclampsia	0 (0)	1 (3.4)	0.185
Prematurity	1 (1.6)	7 (24.1)	0.035
IUGR	1 (1.6)	8 (27.5)	0.023
Abortion	4 (6.7)	4 (13.7)	0.763
In utero death	3 (5.0)	4 (13.7)	0.649
Active disease	0	5(17.2)	0.001

Obstetric complication rates were more frequent in the post-d group than in the pre-d group. There were 5 patients with active disease in the last trimester, 3 of them had stopped their medication, and 2 patients were receiving ineffective treatment. In the group that stopped their medication, there was 1 low birth weight and 2 healthy fetuses. We did not find a statistically significant difference in pregnancy outcomes between age groups.

### Outcomes in different angiographic classification

Obstetric complications of 35 patients according to the Numano classification are shown in Table 3. As seen in the table, type V is the most common type and obstetric outcomes such as preeclampsia, eclampsia, prematurity, low birth weight, abortion, ın utero death, and cesarean section are most common in type V compared to other types. Hypertension occurred in 22 patients, of which 9 were type V,and 6 were type I. Type V had hypertension in 9 of the 14 patients. In hypertensive patients, preeclampsia (*p* = 0.003), prematurity (*p* = 0.036), abortion (*p* = 0.018), in utero death (*p* = 0.128), low birth weight (*p* = 0.250) and cesarean rate (*p* = 0.005) were more frequent than those without hypertension. Renal artery involvement was present in 15 (42.8%) patients. All patients with renal artery involvement had hypertension and all pregnancy complications that occurred in these patients were higher than the other group (*p* = 0.001).

**Table 3:** Obstetric and neonatal outcomes in different angiographic classification

	Patients	Hypertension	Preeclampsia	Eclampsia	Prematurity	IUGR	Abortion	IUD	CSR
	*n*:35	*n*:22	*n*:6	*n*:1	*n*:8	*n*:9	*n*:8	*n*:7	*n*:11
Type I	10	6	0	0	0	0	1	0	1
Type II	7	3	0	0	3	2	2	1	0
Type III	2	2	0	0	0	0	1	1	1
Type IV	2	2	0	0	0	0	0	1	1
Type V	14	9	6	1	5	7	4	4	8

*IUGR* Intrauterine growth restriction**,** *IUD* In utero death, *CSR* Cesarean section rate

## Discussion

TA is a rare chronic inflammatory vascular disease of unknown etiology that primarily affects the aorta and its major branches [1, 16]. Clinical features vary depending on the location and extent of lesions. The type of arterial involvement has an impact on the outcomes. The incidence of preeclampsia, hypertension, and growth restriction in the fetus is found to be higher when the abdominal aorta and renal artery are involved [17]. Partial occlusion of the renal artery causes an increase in renin production, which in turn causes hypertension and decreased blood flow to the uterus and placenta, resulting in fetal growth restriction [17]. Clinical features and arterial involvement patterns show differences in different regions of the world [18]. We will discuss our study results and review previously published reports on pregnancy outcomess in TA patients.

In this study, our results have shown that the main complications associated with TA are new-onset or worsening hypertension for mothers and preeclampsia, abortion, low birth weight prematurity, and in utero death for fetuses. Inconsistencies within the literature exist regarding the report of maternal complications and fetal outcomes associated with TA in distinct studies [6, 19]. Hypertension is the most common maternal complication in women with TA, with incidence rates ranging from 5.3% to 100% [6, 19]. Our study, which used a large sample size, confirmed the previous report with an incidence of 62.9% in our study. Eighteen patients (72%) with high blood pressure were treated with anti-hypertensive medications. Although 7 patients had hypertension, they did not receive any treatment. 15 patients had renal artery involvement. All of these patients developed hypertension and they had higher rates of preeclampsia, low birth weight, prematurity, and cesarean section.

Women with TA who had renal artery involvement were at a significant risk of vascular problems, which could have a severe impact on fetal and maternal outcomes [9]. Our study showed an association between pre-pregnancy renal artery involvement and adverse pregnancy outcomes and maternal complications, consistent with previous research. In addition, the risk of hypertension during pregnancy is increased significantly if the renal arteries are affected before pregnancy. Therefore, it is important to treat renal artery stenosis effectively before pregnancy. This can reduce the risk of adverse pregnancy outcomes.

Our study found only six TA patients (6.8%) who developed preeclampsia and one patient eclampsia in striking contrast to the literature report of up to 75.9% in the preeclampsia/eclampsia rate [6, 20–22]. The low incidence of eclampsia/preeclampsia in our study may be due to the effective management of hypertension and TA disease before and following pregnancy. All of the patients with preeclampsia had type V arterial involvement. Also, 8 out of 9 patients with low birth weight were type V. Type V involvement pattern is a risk factor for hypertension as well as for preeclampsia, low birth weight, and prematurity.

In a meta-analysis of 505 pregnancies among 373 TA patients, a rate of 12% for miscarriages and a rate of 7% for therapeutic abortions were observed [23]. Therapeutic abortions may be performed to terminate a pregnancy when the mother's life is at risk or if the fetus has abnormalities affecting major organ systems and is not expected to survive after birth [24]. It may be considered in periods when TA is active, there was no therapeutic abortion in our study. In our study, spontaneous abortions occurred in 9.0% of pregnancies of patients with TA. A total of 22.4% of married women had a spontaneous abortion, and 15% had induced abortion in the general female population in Turkey [25] the rate in TA patients was the lowest. This may be the outcome of a current illness just before conception.

We had a 31.4% CS rate in our cohort, mostly for obstetric indications. Compared with some previous reports, the cesarean section rate was lower [17, 26]. In severe disease categories such as type V arterial involved, women were more likely to have CS (Table 3). This is because the more severe angiographic types are more likely to have obstetric complications. Intrapartum variations in blood pressure and heightened cardiac output may exacerbate extant maternal issues associated with TA [17, 26, 27]. The existing uteroplacental insufficiency may worsen and result in fetal compromise.

IUGR may arise due to compromised blood flow to the placenta or stenosis or occlusion of the renal artery, resulting in heightened renin production and subsequent elevation of blood pressure. This has detrimental effects on placental blood circulation [21]. Indeed, from all 9 women with IUGR, 3 had abdominal aortitis, and 7 had renal artery stenosis. In concordance with others, we found an association between infra diaphragmatic artery involvement and worsening maternal hypertension during pregnancy.

In some studies, it has been shown that the risk of premature birth is increased in TA pregnancies [19]. In our study, an increase was found in the rate of prematurity in pregnancies diagnosed with post-TA compared to pre-TA (24.1% vs 1.6%, *p* = 0,035).

Disease activity of TA could be associated with a poor pregnancy outcome [8, 21, 28]. In our experience, active disease (NIH score ≥ 2) was observed in 5 pregnancies (5.6%) whereas inactive disease (NIH score ≤ 1) was observed in 83 pregnancies (94.3%). No association was found between TA activity before or during pregnancy and adverse obstetric complications. 5 patients had active disease in the third trimester, 3 of them had stopped their medications, and 2 patients were receiving ineffective treatment. In the group who stopped their medications, there was 1 low birth weight and 2 healthy fetuses.

Hypertension was seen in 12 of the pre-d patients, 7 of whom had type V involvement. It was seen in 10 of the post-d patients, 8 of whom had type V involvement. Preeclampsia and eclampsia were not seen in pre-diagnosis pregnancies, while they were seen in post-diagnosis pregnancies and all of them had type V involvement. We would like to point out that the most important risk factor for complications in our study was the type V arterial involvement pattern. This shows us that determining the angiographic arterial involvement pattern before pregnancy is very important. Those who were previously type I, II, or III should also be investigated for type IV and V before pregnancy.

There are certain limitations in our study. Our analysis was performed as a retrospective review. TA is a rare condition, which has led to a relatively limited sample size in our study. In addition, there is the possibility of a bias in the memory, since the patients were asked about facts from the past. The lack of data on disease activity before, during and after pregnancy was also a limitation. CRP and ESR, commonly used as indicators of inflammation in the NIH activity score, may be moderately elevated during pregnancy, independent of disease activity. Another limitation the absence of a control group of age-matched healthy pregnant women prevents an accurate estimation of the different frequencies of adverse obstetrical events.

## Conclusion

In this study, we observed that hypertension was seen in all arterial involvement patterns, but more frequently in type V involvement, and also that the rates of preeclampsia, abortion, low birth weight and cesarean section were low in those who had pregnancy before the diagnosis, while these complications increased after the diagnosis. We think that this is mainly associated with the type V arterial involvement pattern. These findings indicate that women with TA are at higher risk of adverse pregnancy outcomes. Thus, more intense follow-up along with a multidisciplinary approach is needed for these patients to reach an optimal delivery.

## Data Availability

Data are available upon reasonable request to the corresponding author.
